# Weight gain, poor mental health and increased sedentary hours among Malaysian adults during the COVID-19 pandemic

**DOI:** 10.1177/02601060231164434

**Published:** 2023-03-20

**Authors:** Guo Fu Ng, Shi-Hui Cheng

**Affiliations:** School of Biosciences, Faculty of Science and Engineering, 69861 University of Nottingham Malaysia, Semenyih, Selangor, Malaysia

**Keywords:** Weight gain, anxiety, depression, stress, lifestyle, sedentary, COVID-19

## Abstract

**Background:**

The Movement Control Orders (MCO) in Malaysia due to the COVID-19 pandemic had a profound impact on the lifestyle behaviours, weight changes, and mental health of the population.

**Aim:**

To determine the changes in physical activity, sedentary behaviour, body weight status and mental health status among Malaysian adults before and during the pandemic.

**Methods:**

A total of 338 Malaysian adults participated in this cross-sectional online study. Sociodemographic and anthropometric data were self-reported. Physical activity and sedentary behaviour were assessed using International Physical Activity Questionnaire-Short Form (IPAQ-SF) while the Perceived Stress Scale (PSS-10), Patient Health Questionnaire (PHQ-9) and Generalised Anxiety Disorder Assessment (GAD-7) were used to examine stress, depression and anxiety, respectively. All statistical analysis was performed using SPSS version 28.0.

**Results:**

The results showed an average weight gain of 0.6 kg among the participants with 45.5% of them experiencing weight gain. In addition, sedentary behaviour (*p* < 0.001), PSS-10 score (*p* < 0.001), PHQ-9 score (*p* = 0.002) and GAD-7 score (*p* = 0.001) were significantly increased during the COVID-19 pandemic whereas the level of physical activity was significantly decreased (*p* = 0.003) during the pandemic. Weight changes during the pandemic were found to be associated with age, sedentary hours, and PHQ-9 score. Through binary logistic regression, sedentary hours (AOR = 1.068, 95% CI = 1.002–1.139, *p* = 0.043) were identified to be a risk factor for weight gain during the pandemic.

**Conclusion:**

The findings suggested that public health interventions to prevent weight gain should focus on strategies to increase physical activity for sedentary lifestyles.

## Introduction

Since the outbreak of coronavirus disease 2019 (COVID-19) caused by SARS-CoV-2 in Wuhan, China, it has spread rapidly across the globe. As part of the measures to halt the spread of the disease, nationwide lockdowns, known as Movement Control Orders (MCO) have been enforced in Malaysia along with stringent Standard Operating Procedures (SOPs) such as social distancing, home confinement and temporary closure of unnecessary businesses. The SOPs in place have had an impact on everyone's lifestyle since outdoor activities are restricted (di Renzo et al., 2020). Physical inactivity is expected to deteriorate compared to pre-COVID-19, as remote learning and smart working have emerged as new norms. Moreover, the suspension of social activities and extended home confinement would allow more time for TV and most likely induce boredom, which is associated with an increased intake of energy-dense snacks that most people would treat as a way to escape boredom (di Renzo et al., 2020). Ultimately, physical inactivity and a sedentary lifestyle would have a detrimental synergistic effect on the weight status among adults (di Renzo et al., 2020).

Aside from altering people's lifestyle, adjournment of social activities and prolonged isolation due to COVID-19 would eventually lead to stress, anxiety and depression in the public thereby further deteriorating the mental health of the population (Al-Musharaf et al., 2021). The association between stress and overeating has been thoroughly researched, whereby those under stress are more likely to crave ‘comfort foods’ which contain a high amount of sugar (Husain and Ashkanani, 2020). Furthermore, the unpleasant experience of prolonged isolation also contributes to the physiological response of seeking reward and satisfaction, resulting in excess food intake and a positive energy balance (di Renzo et al., 2020).

Altogether, physical inactivity, sedentary lifestyle and deteriorated mental health have effects on weight changes during the pandemic. Although the SOPs has been gradually loosened, the ongoing mutation of COVID-19 and its continually high rate of transmission suggest that the pandemic will not end with the development of vaccines. Therefore, this study aims to determine the changes in lifestyle, mental health, and body weight status among Malaysian adults before and during COVID-19.

## Materials and methods

### Study design and ethics approval

A cross-sectional study was conducted among Malaysian adults before and during COVID-19. Data before COVID-19 were collected from September 2019 through means of recalling, whereas data during COVID-19 were collected from November 2021 to February 2022. A research ethics committee of the university approved this study (Ethics approval ID: NGF061121).

### Subjects and recruitment procedures

The sample size was computed with the formula $n=\frac{z^{2}pq}{d^{2}}$, where *n* = desired sample size, *z* = confidence level, *p* = prevalence, *q* = 1–*p* and *d* = margin of error (Sharma et al., 2019). Based on the study by Zheng et al. (2020), physical inactivity increased by 70% during the COVID-19 pandemic. Hence, the sample size required in this study was 323 at a 95% confidence interval.

Snowball sampling was used to recruit participants. Malaysian adults aged 18 and above were invited to participate in this study via multiple communication platforms including email, WhatsApp, Facebook, and Instagram. Adults who had COVID-19 infection at the time of completing the survey, having any mental health issues, physical disabilities or chronic diseases and pregnant women were excluded from this study.

### Data collection

The questionnaire was made up of three sections: (1) sociodemographic and anthropometric information; (2) physical activity and sedentary behaviour; and (3) mental health status. All responses were self-reported by the participants.

#### Sociodemographic and anthropometric information

Sociodemographic information including gender, age, highest education level, ethnicity, monthly household income, employment status, smoking status and lockdown status were collected. For anthropometric measurement, the height and weight of the participants before the pandemic were self-reported by the participants. On the other hand, the body weight of the participants during the pandemic was self-measured with a bathroom scale with minimal clothes, and shoes off. Body weight changes were reflected by the difference before and during the pandemic and were further categorised into ‘decreased weight’, ‘no change’ or ‘increased weight’. Additionally, body mass index (BMI) was derived by dividing body weight (kg) with the square of height (m) thereafter classified based on the Asia-Pacific cutoff points whereby below 18.5, 18.5–22.9, 23.0–27.4, and above 27.5 kg/m^2^ represents underweight, normal weight, overweight, and obesity, respectively, (Lim et al., 2017).

#### Physical activity and sedentary behaviour

The physical activity level was examined by using the International Physical Activity Questionnaire-Short Form (IPAQ-SF). The questionnaire consisted of seven questions that aimed to gather data on the duration of sedentary behaviour, walking, and moderate, and vigorous physical activities by the participants for the past seven days (International Physical Activity Questionnaire, 2004). The weekly metabolic equivalent (MET) minute was computed by multiplying the MET factor allocated for each activity (walking = 3.3 MET, moderate physical activity = 4.0 MET, vigorous physical activity = 8.0 MET) with the duration spent on the activity and the number of days the activity was carried out in a week. To classify the participants according to how active they were, MET minutes per week were computed by summing up the weekly MET minutes from walking, moderate, and vigorous physical activity. Based on the WHO recommendation, participants who achieved 600 MET-minutes/week and above were classified as physically active whereas those who obtained less than 600 MET-minutes/week were classified as physically inactive. Duration spent on sedentary behaviour including sitting and lying down was addressed by asking the participants to list down the duration they spent sitting on a weekday for the past seven days in the final question. Subsequently, participants were divided into two groups of sedentary and non-sedentary, with an 8-hour cutoff time for sedentary behaviour (Tan et al., 2021).

#### Perceived stress questionnaire

The Perceived Stress Scale (PSS-10) was used to evaluate the stress level among the participants for the past one month during the pandemic. Each aspect was based on a scale of zero to four whereby a higher score indicated less severe stress in questions 4, 5, 7 and 8 but more severe stress for the rest of the questions (Cohen et al., 1983). The final score ranging from 0 to 40 was obtained by summing up the score from all 10 questions. Participants were then classified into three separate groups of slightly (0–13), moderately (14–26) and severely stressed (27–40) based on their scores.

#### Depression scale

Patient Health Questionnaire (PHQ-9) was used to assess the level of depression before and during the pandemic (Kroenke et al., 2001). The questionnaire consisted of nine items that required participants to answer on how frequently they were bothered by the problems for the past two weeks. The responses to the questions were corresponded to a score of zero to three. The points scored in each item were added up to obtain the final score of 0–4, 5–9, 10–14, 15–19 and 20–27 indicating minimal, mild, moderate, moderately severe and severe depression, respectively.

#### Anxiety scale

The Generalized Anxiety Disorder (GAD-7) (Spitzer et al., 2006) was used to assess the level of anxiety before and during the pandemic. It consisted of seven questions which required the participants to report how frequently they felt the feelings for the past two weeks. The score was allocated and summed up to obtain the final score. Different ranges of final scores at 0–4, 5–9, 10–14 and 15–21 reflected minimal, mild, moderate and severe anxiety, respectively.

### Statistical analysis

Data collected was analysed by using IBM SPSS Statistics version 28.0 (IBM Corp., Armonk, NY, USA). Continuous variables were expressed as mean and standard deviation (SD) while categorical variables were presented as frequencies in percentage (%). The differences between the means of continuous variables before and during the pandemic were determined using paired *t*-tests. The association between lifestyle behaviours and weight change as well as mental health and weight change were examined using the chi-square test for categorical variables and ANOVA for continuous variables. Fisher's exact test was performed on a variable with a frequency of less than 5. All variables with *p* < 0.25 in the chi-square test and ANOVA test were included in the binary logistic regression to determine the factors associated with body weight changes. The reference group used in the binary logistic regression was weight loss or no changes in weight. The *p*-value was considered statistically significant at *p* < 0.05.

## Results

### Changes in weight change-related parameters before and during COVID-19

Table 1 presents the changes in body weight-related parameters before and during the COVID-19 pandemic. Overall, there was a significant weight gain in the participants from 63.7 ± 16.4 to 64.3 ± 16.9 kg during the COVID-19 pandemic. Besides, a significant increase in the BMI of the participants from 24.1 ± 5.5 to 24.3 ± 5.6 kg/m^2^ was found during the COVID-19 pandemic. The level of physical activity has dropped significantly during the COVID-19 pandemic. Conversely, the mean sedentary hours of the participants were significantly increase from 6.4 ± 3.6 to 7.1 ± 3.8 h during the pandemic. In terms of mental health, all the parameters have increased significantly as reflected by the score obtained in PSS-10, PHQ-9 and GAD-7, respectively.

**Table 1.** Comparison of weight change-related parameters before and during COVID-19.

Parameters	Mean ± SD		*p*-value
	Before COVID-19 (n = 338)	During COVID-19 (n = 338)	
**Anthropometry parameters**			
Body weight (kg)	63.7 ± 16.4	64.3 ± 16.9	**0.011**
BMI (kg/m^2^)	24.1 ± 5.5	24.3 ± 5.6	**0.017**
**Lifestyle parameters**			
Physical activity (MET-minutes/week)	2986.3 ± 3433.0	2600.0 ± 3324.5	**0.003**
Sedentary behaviour (hour/day)	6.4 ± 3.6	7.1 ± 3.8	**<0.001**
**Mental health parameters**			
PSS-10 score	18.4 ± 5.0	19.5 ± 5.9	**<0.001**
PHQ-9 score	5.7 ± 5.2	6.5 ± 6.2	**0.002**
GAD-7 score	4.9 ± 4.8	5.6 ± 5.4	**0.001**

BMI: body mass index; MET: metabolic equivalent; PSS-10: Perceived Stress Scale; PHQ-9: Patient Health Questionnaire; GAD-7: Generalised Anxiety Disorder Assessment.

Paired *t*-test was performed with a significance level of *p* < 0.05.

### Changes in body weight and BMI status during the COVID-19

Table 2 shows the overall changes in body weight and BMI status during COVID-19. Generally, the change in body weight during the COVID-19 pandemic was an average weight gain of 0.6 ± 4.2 kg with those in the underweight category experiencing the largest weight gain at 0.9 ± 2.6 kg. Before the COVID-19 outbreak, 38.8% of the participants had normal BMI, 29.3% were overweight, 20.7% were obese and 11.2% were classified as underweight. During the COVID-19 pandemic, about half of them experienced weight gain (45.5%) while 32.0% of them lost weight and 22.5% with unchanged body weight.

**Table 2.** Changes in body weight and body mass index (BMI) status during COVID-19.

Parameters	Total (n = 338)	BMI status before COVID-19, n (%)				*p*-value
		Underweight (n = 38)	Normal (n = 131)	Overweight (n = 99)	Obesity (n = 70)	
**Body weight changes (kg)**	0.6 ± 4.2	0.9 ± 2.6	0.4 ± 3.0	0.8 ± 4.3	0.4 ± 6.2	0.860
Decreased	108 (32.0)	10 (26.3)	38 (29.0)	32 (32.3)	28 (40.0)	0.617
No change	76 (22.5)	7 (18.4)	32 (24.4)	24 (24.3)	13 (18.6)	
Increased	154 (45.5)	21 (55.3)	61 (46.6)	43 (43.4)	29 (41.4)	
**BMI status during COVID-19**						** < 0.001**
Underweight	30 (8.9)	25 (65.8)	5 (3.8)	0	0	
Normal	137 (40.5)	13 (34.2)	113 (86.3)	11 (11.1)	0	
Overweight	95 (28.1)	0	13 (9.9)	77 (77.8)	5 (7.1)	
Obesity	76 (22.5)	0	0	11 (11.1)	65 (92.9)	

ANOVA was performed on continuous variables and the chi-square test was performed on categorical variables. Fisher's exact test was performed on a variable with a frequency of less than 5. The significance level was set at *p* < 0.05.

Many of the participants experienced significant changes in BMI status whereby 34.2% of underweight participants and 11.1% of overweight participants managed to achieve normal BMI during the pandemic. However, 3.8% and 9.9% of those with normal BMI before the pandemic had developed to become underweight and overweight, respectively, during the pandemic. Besides, 11.1% of overweight participants gained weight to become obese, while 7.1% of the obese participants lose weight and returned to the overweight group during the pandemic.

### Associations between sociodemographic characteristics, lifestyle behaviours and mental health with body weight changes during the COVID-19

Table 3 shows the sociodemographic characteristics of the participants, with a mean age of 33.7 ± 14.0 years, and their associations with body weight changes during COVID-19. A majority of the participants were female (68.6%) and had completed an undergraduate (45.9%) as their highest education level. For ethnicity, the majority of the participants were of Chinese origin (79.6%), while 18.0% were Bumiputera and Malay, 1.2% were Indian and 1.2% were other from other minor ethnicities. More than half (53.2%) were from households with a monthly income of RM5000–RM9999 (∼ USD1185‒2371). Most of the participants were non-smokers (96.7%) and were under National Recovery Plan (NRP) Phase 3 (73.4%). During the COVID-19 pandemic, the majority of them remained physically active (71.9%) while 54.4% were non-sedentary with 7.1 ± 3.8 h, on average, of sedentary behaviour in a day. About three-quarters of the participants had a moderate stress level (73.4%) with a mean PSS-10 score of 19.5 ± 5.9. On the other hand, about half of the participants had minimal depression (50.5%) and minimal anxiety (50.6%) with a mean PHQ-9 score and mean GAD-7 score of 6.5 ± 6.2 and 5.6 ± 5.4, respectively.

**Table 3.** Associations between sociodemographic, lifestyle behaviours and mental health with body weight changes during COVID-19.

Parameters	Total (n = 338)	Weight changes			*p*-value
		Decreased (n = 108)	No change (n = 76)	Increased (n = 154)	
	n (%)/Mean ± SD	n (%)/Mean ± SD	n (%)/Mean ± SD	n (%)/Mean ± SD	
**Age**	33.7 ± 14.0	34.4 ± 14.6	37.3 ± 14.0	31.5 ± 13.2	**0**.**010**
**Gender**					0.430
Male	106 (31.4)	29 (26.9)	24 (31.6)	53 (34.4)	
Female	232 (68.6)	79 (73.1)	52 (68.4)	101 (65.6)	
**Highest education level**					0.910
Secondary or lower	24 (7.1)	6 (5.6)	4 (5.3)	14 (9.1)	
A-levels/diploma	68 (20.1)	21 (19.4)	16 (21.0)	31 (20.1)	
Undergraduate	155 (45.9)	53 (49.1)	35 (46.1)	67 (43.5)	
Postgraduate	91 (26.9)	28 (25.9)	21 (27.6)	42 (27.3)	
**Ethnicity**					0.411
Chinese	269 (79.6)	93 (86.1)	56 (73.7)	120 (77.9)	
Indian	4 (1.2)	1 (0.9)	1 (1.3)	2 (1.3)	
Bumiputera/Malay	61 (18.0)	13 (12.1)	17 (22.4)	31 (20.1)	
Others	4 (1.2)	1 (0.9)	2 (2.6)	1 (0.7)	
**Monthly household income**					0.164
Below RM2500	79 (23.4)	27 (25.0)	17 (22.4)	35 (22.7)	
RM2500 – RM4999	79 (23.4)	19 (17.6)	25 (32.9)	35 (22.7)	
RM5000 – RM9999	129 (38.1)	48 (44.4)	26 (34.2)	55 (35.7)	
RM10,000 and above	51 (15.1)	14 (13.0)	8 (10.5)	29 (18.9)	
**Employment status**					0.163
Employed – Full-time	163 (48.2)	53 (49.1)	41 (53.9)	69 (44.8)	
Employed – Part-time	8 (2.4)	1 (0.9)	2 (2.6)	5 (3.2)	
Self-employed	15 (4.4)	3 (2.8)	6 (7.9)	6 (3.9)	
Retired	10 (3.0)	4 (3.7)	4 (5.3)	2 (1.3)	
Student/not employed	142 (42.0)	47 (43.5)	23 (30.3)	72 (46.8)	
**Smoking status**					0.787
Smoker	11 (3.3)	4 (3.7)	3 (3.9)	4 (2.6)	
Non-smoker	327 (96.7)	104 (96.3)	73 (96.1)	150 (97.4)	
**Lockdown status**					0.718
National Recovery Plan (NRP) Phase 3	248 (73.4)	80 (74.1)	58 (76.3)	110 (71.4)	
National Recovery Plan (NRP) Phase 4	90 (26.6)	28 (25.9)	18 (23.7)	44 (28.6)	
**MET-minutes/week**	2600.0 ± 3324.5	2408.2 ± 2962.2	3161.1 ± 4562.9	2457.7 ± 2792.7	0.246
**Physical activity status**					0.390
Physically active	243 (71.9)	76 (70.4)	51 (67.1)	116 (75.3)	
Physically inactive	95 (28.1)	32 (29.6)	25 (32.9)	38 (24.7)	
**Sedentary hours/day**	7.1 ± 3.8	6.8 ± 3.9	6.0 ± 3.6	7.8 ± 3.8	**0**.**003**
**Sedentary behaviour**					0.087
Sedentary	154 (45.6)	45 (41.7)	29 (38.2)	80 (51.9)	
Not sedentary	184 (54.4)	63 (58.3)	47 (61.8)	74 (48.1)	
**PSS-10 score**	19.5 ± 5.9	19.6 ± 6.1	19.0 ± 5.2	19.6 ± 6.2	0.729
**Stress level**					0.468
Low	46 (13.6)	15 (13.9)	7 (9.2)	24 (15.6)	
Moderate	248 (73.4)	78 (72.2)	62 (81.6)	108 (70.1)	
High	44 (13.0)	15 (13.9)	7 (9.2)	22 (14.3)	
**PHQ-9 score**	6.5 ± 6.2	6.6 ± 5.9	4.9 ± 6.0	7.3 ± 6.3	**0**.**019**
**Depression level**					**0**.**035**
Minimal	171 (50.5)	54 (50.0)	52 (68.4)	65 (42.2)	
Mild	72 (21.3)	22 (20.4)	10 (13.2)	40 (26.0)	
Moderate	52 (15.4)	19 (17.6)	7 (9.2)	26 (16.9)	
Moderately severe	35 (10.4)	12 (11.1)	5 (6.6)	18 (11.7)	
Severe	8 (2.4)	1 (0.9)	2 (2.6)	5 (3.2)	
**GAD-7 score**	5.6 ± 5.4	5.6 ± 5.2	4.7 ± 5.9	6.1 ± 5.3	0.166
**Anxiety level**					0.068
Minimal	171 (50.6)	57 (52.8)	48 (63.1)	66 (42.9)	
Mild	96 (28.4)	31 (28.7)	12 (15.8)	53 (34.4)	
Moderate	44 (13.0)	11 (10.2)	11 (14.5)	22 (14.3)	
Severe	27 (8.0)	9 (8.3)	5 (6.6)	13 (8.4)	

RM, Ringgit Malaysia (1 USD = RM4.19, as of 20 March 2022); MET: metabolic equivalent; PSS-10: Perceived Stress Scale; PHQ-9: Patient Health Questionnaire; GAD-7: Generalised Anxiety Disorder Assessment.

ANOVA was performed on continuous variables and the chi-square test was performed on categorical variables. The significance level was set at *p* < 0.05.

Among these, the parameters that were found to have significant associations with weight changes during the COVID-19 pandemic were age, sedentary hours and PHQ-9 score. Participants who had gained weight during the pandemic were significantly younger (31.5 ± 13.2 years) than those who had decreased (34.4 ± 14.6 years) or remained the same weight (37.3 ± 14.0 years). Additionally, participants who gained weight during the COVID-19 pandemic had significantly longer sedentary hours (7.8 ± 3.8 h) than those who lost weight (6.8 ± 3.9 h) or unchanged body weight (6.0 ± 3.6 h). Similarly, PHQ-9 scores were found to be significantly higher among participants with weight gain (7.3 ± 6.3) than participants with decreased (6.6 ± 5.9) or maintained body weight (4.9 ± 6.0).

### Risk factors associated with body weight changes during COVID-19

Table 4 demonstrates the risk factors associated with body weight changes during COVID-19. Among the factors assessed, the sedentary hour was found to be significantly associated with weight gain during COVID-19. Participants were found to be 1.068 times more likely to gain weight during the COVID-19 pandemic for every 1 h increase in sedentary behaviour (AOR = 1.068, 95% CI = 1.002–1.139, *p* = 0.043). No significant associations were found between age, monthly household income, employment status, MET-minutes/week, PHQ-9 score and GAD-7 score with weight gain during the COVID-19.

**Table 4.** The odds ratio for possible risk factors that may affect body weight changes during COVID-19.

Parameters	Weight gain during COVID-19	
	Adjusted odds ratio (95% CI)	*p*-value
**Age**	0.981 (0.961–1.001)	0.058
**Monthly household income**		
< RM2500	1	
≥ RM2500	1.187 (0.942–1.496)	0.145
**Employment status**		
Full-time employment	1	
Non-full-time employment	0.937 (0.797–1.101)	0.429
**MET-minutes/week**	1.000 (1.000–1.000)	0.843
**Sedentary hours**	1.068 (1.002–1.139)	**0**.**043**
**PHQ-9 score**	1.034 (0.969–1.103)	0.310
**GAD-7 score**	0.984 (0.915–1.057)	0.652

CI, confidence interval; MET: metabolic equivalent; PHQ-9: Patient Health Questionnaire; GAD-7: Generalised Anxiety Disorder Assessment.

Binary logistic regression was performed with all confounding variables of *p* < 0.25 during the previous chi-square and ANOVA tests. The significance level was set at *p* < 0.05. Reference group for dependent variable: weight loss or no changes in weight (n = 184); weight gain (n = 154).

## Discussion

This study revealed 45.5% of the participants had an average weight gain of 0.6 ± 4.2 kg during the pandemic. This finding indicated a relatively higher proportion of the population experiencing weight gain compared to the previous studies, which may be explained by the higher percentage of participants who reported a moderate level of stress. For instance, weight gain was reported among 12.8% of the Spanish population (Rodríguez-Pérez et al., 2020), 33.6% (Drywień et al., 2020) and 38% (Reyes-Olavarría et al., 2020) of the Polish and Chilean population. These variations are probably due to the implementation of different restrictive measures as well as the diverse cultures and dietary habits among different countries (Al-Musharaf et al., 2021). Nonetheless, the average weight gain found in this study is consistent with the weight gain of 0.62 kg reported in the USA (Bhutani et al., 2021) and 0.5 kg reported in China (Zhu et al., 2021). Interestingly, a much higher weight gain of 1.5 kg was reported in Italy (Pellegrini et al., 2020) while 3 kg of weight gain was reported among the Polish population (Sidor and Rzymski, 2020). On the other hand, weight loss was reported among 32% of the participants in this study which was comparable to the percentage previously reported in Malaysia (Chin et al., 2022) and China (Xia et al., 2020).

Weight gain was observed across all BMI categories in this study, with those in the underweight category experiencing the greatest weight gain. The changes in mean MET-minutes per week before and during the COVID-19 by BMI category are shown in Appendix 1. In addition, sedentary hours increased significantly in underweight group before and during COVID-19 (*p* = 0.026) (Appendix 1). Our findings are in line with previous studies, which have suggested that individuals with lower BMI were more likely to engage in physical activities and live a healthier lifestyle before the pandemic (He et al., 2020). However, because access to physical activity resources was very limited under the lockdown circumstances, a reduction in physical activity was more obvious among those with a lower BMI (Alshahrani et al., 2022). Additionally, individuals with lower BMI were less bothered by excess body weight, therefore, making them less aware of weight control and more prone to weight gain (He et al., 2020).

Among the obese, a surprisingly high percentage (40.0%) of them lost weight during the pandemic. This finding can be explained by the greater awareness among those with higher BMI, who are more likely to control their food intake and increase physical activity. Before the pandemic, they were unable to effectively reduce their body weight due to frequent socializing and drinking as part of their work regimes (He et al., 2020). However, the circumstances under lockdown were ideal for them to lose weight as they now have more time to exercise at home compared to before the pandemic where they only exercise occasionally (di Renzo et al., 2020).

The change in BMI status during the pandemic was significant (*p* < 0.001), with 9.9% of those with normal BMI becoming overweight and 11.1% of those overweight developing obesity. An increase in overweight and obese individuals by 4.8% and 3.3%, and 4.8% and 5.1% were reported in China (Liu et al., 2021) and Saudi Arabia (Alshahrani et al., 2022), respectively. The discrepancy between the findings can be explained by the timing of data collection since the previous studies were conducted soon after the lockdown was implemented while data in the present study was collected after the lockdown was implemented for an extended period. The overall increasing trend in body weight during the pandemic, resulting in an increase in overweight and obese individuals, is alarming and should be addressed when it comes to policy making.

Participants who gained weight during the pandemic were significantly younger (*p* = 0.010). Further statistical analysis revealed that the mean MET-minutes per week reduced significantly in the 18–30 younger age group before and during COVID-19 (*p* = 0.007) (Appendix 1). In addition, sedentary hours increased significantly in the 18–30 age group before and during COVID-19 (*p* < 0.001). This can be explained by previous studies which stated that the decline in physical activity was greater among younger adults (Curtis et al., 2021). Besides, over-eating to cope with stress during the pandemic was noted to be prevalent among young adults (Cheng and Kamil, 2020; Cheng and Wong, 2021). This finding is of clinical importance as weight gain among youths can substantially increase over the years and lead to comorbidities.

The current study revealed a significant decrease in physical activity (*p* = 0.003) from before the pandemic (2986.3 MET-minutes/week) to during the pandemic (2600.0 MET-minutes/week) due to the lockdown. The average physical activity during the pandemic found in this study is comparable with the median score of 2826.0 MET-minutes/week previously reported among Malaysian students (Tan et al., 2021). Despite the fact that overall physical activity decreased during the pandemic, a relatively large proportion of the population (71.9%) remained physically active during the lockdown, which is consistent with previous studies that found 76.0% (Chin et al., 2022) and 79.6% (Tan et al., 2021) of Malaysians to be physically active during the pandemic. Additionally, the proportion of participants classified to be physically inactive (28.1%) in this study is consistent with WHO statistics, which revealed that 23% of adults worldwide were physically inactive (World Health Organization, 2022). The reduction in physical activity during the pandemic in the present study was in line with studies in Kuwait (AlMughamis et al., 2021), Lithuania (Kriaucioniene et al., 2020) as well as Chile (Reyes-Olavarría et al., 2020). Conversely, increased physical activity was observed in a British cohort study (Bann et al., 2021) and an Italian survey (di Renzo et al., 2020). The discrepancy can be explained by the different periods of data collection and different restrictive measures being implemented.

On the contrary, this study found a significant increase in sedentary behaviour (*p* < 0.001), with an average of 7.1 h spent on screens and sitting down during the pandemic. This finding contradicts the findings reported by Tan et al., who found that Malaysian university students spent 9.2 h on sedentary behaviour. The longer sedentary hour reported by Tan et al. may be explained by the previous study focused on university students alone, who were expected to commit to longer screen time due to online classes. Besides, almost half of the participants (45.6%) were classified as sedentary in this study. A similar trend was observed in Kuwait (Husain and Ashkanani, 2020) and Saudi Arabia (Alshahrani et al., 2022), where 43.6% and 36.2% of the population, respectively, were found to be sedentary during the pandemic. Although smart working could be a factor, other platforms including TikTok and Netflix were reported to be the main reason for increased screen time.

More importantly, the present study found that participants are 1.068 times more likely to gain weight during the COVID-19 pandemic for every 1 h spent on sedentary behaviour. This is comparable to the previous research, which concluded that sedentary behaviour including watching TV and using the computer was a risk factor for weight gain (Ashdown-Franks et al., 2019). Similarly, among Arabian women, there was a significant positive association between weight change and a sitting time (Al-Musharaf et al., 2021). In another study, sedentary time was associated with an average weight gain of 0.53 kg in males and 0.46 kg in females (Liu et al., 2021). This association can be explained by increased snacking frequency, which leads to over-consumption of the next meal, eventually resulting in excess weight (Rodríguez-Pérez et al., 2020). Moreover, studies have shown that sedentary behaviour increased the risk of weight gain not only among obese individuals (Pellegrini et al., 2020), but also among those with normal BMI (Reyes-Olavarría et al., 2020).

The present study found that the stress level among Malaysian adults increased significantly (*p* < 0.001) during the pandemic, with 73.4% of the population experiencing moderate stress. This is consistent with previous studies indicating that the pandemic has led to an increase in stress levels across different populations. The symptoms of mental distress were reported to be significantly higher in the USA (Ettman et al., 2020) and the UK (Pierce et al., 2020) during the pandemic than in pre-COVID. Increased stress is anticipated under the pandemic circumstances, particularly during the early phase of the lockdown, due to the presence of a novel and unknown virus (Al-Musharaf et al., 2021). Besides, constantly reading or hearing about the pandemic under restrictions due to the lockdown as well as the fear of disease and death can further deteriorate the stress level within a population (di Renzo et al., 2020).

Depression was observed to deteriorate significantly during the pandemic (*p* = 0.002) in the current study, with almost half of the population suffering from depression of varying degrees. This is in line with an Australian study that reported at least a doubling of depressive symptoms among 15,000 respondents (Curtis et al., 2021). Besides, our study reported a positive association between PHQ-9 score and weight change, which is corroborated by a previous study that indicated a significant association between depression and increased weight (Pellegrini et al., 2020). Additionally, depression score was reported to be independently associated with weight change whereby a 0.14 kg weight gain was observed in participants with a greater increase in depression score (Liu et al., 2021). To mitigate the negative experience of prolonged isolation and confinement, people became more susceptible to ‘food craving’ which often overrides the signals of satiety and hunger. Despite the advice to consume food containing high fat and sugar in moderation, chocolate and crisps were reported to be the most consumed snacks during the pandemic (Husain and Ashkanani, 2020). Ultimately, the over-consumption of ‘comfort food’ due to depression during the pandemic may contribute to an increased risk of weight gain and obesity.

Anxiety showed a similar trend, with the GAD-7 score increased significantly (*p* = 0.001) during the pandemic, and 49.4% of the participants suffered from anxiety. This finding was in line with early studies that observed a higher prevalence of anxiety under lockdown circumstances in New Zealand (Sibley et al., 2020) and Spain (Rodríguez-Rey, Garrido-Hernansaiz and Collado, 2020). The deterioration of mental health during the pandemic can induce behavioural changes (di Renzo et al., 2020) as well as affect food choices (Cheng and Wong 2021). Besides, emotional eating associated with higher levels of stress and anxiety could lead to excessive intake of comfort food containing high amount of sugar and fat (Husain and Ashkanani, 2020). Furthermore, Malaysian adults reported an increased consumption of sugar-sweetened beverages (Cheng and Lau, 2022) and decreased intake of fruits and vegetables (Lo et al., 2022) during COVID-19. These findings suggest that stress, depression, and anxiety can synergistically cause adverse effects on body weight during the pandemic due to poor dietary choices. Hence, the importance of mental health during the pandemic should be emphasized.

The present study has several limitations that must be acknowledged. First, all data collected were self-reported by the participants. These data may be subjected to recall bias and over- and underestimation may be present to a certain degree. Secondly, an online questionnaire was used in this study and was disseminated through online platforms in which participants were recruited through the snowball sampling method. Therefore, oversampling of a particular group including a higher proportion of female gender and Chinese ethnicity in this study should be highlighted. Besides, dietary intake which could be a confounding factor in weight changes during the pandemic was not assessed in this study. Hence, a more inclusive longitudinal study assessing all possible risk factors and a larger sample size should be carried out to accurately determine the association between lifestyle behaviours, mental health, and weight change during the COVID-19 pandemic. Nonetheless, this study presented an insight into the changes in lifestyle behaviours and mental health along with their impacts on weight changes among Malaysian adults during the pandemic.

## Conclusion

In conclusion, weight gain was observed across all BMI categories among Malaysian adults during the COVID-19 pandemic. During the pandemic, physical inactivity and sedentary behaviour increased significantly, while the prevalence of stress, depression and anxiety has significantly heightened among Malaysian adults. In particular, increased sedentary behaviour was found to be associated with an increased risk of weight gain during the pandemic. While the lockdown has caused enormous changes in lifestyle and mental health leading to an increased risk of weight gain, these findings provide a preliminary insight for the development of nutrition and lifestyle guidelines to promote healthy body weight and well-being during the pandemic. Therefore, strategies to increase physical activity such as planning an exercise regimen should be implemented to reduce sedentary hours and prevent weight gain among Malaysian adults.

## Appendix 1

**Table A1.** Changes in MET-minutes/week and sedentary hours/day before and during COVID-19 by BMI category and age group.

	MET-minutes/week before COVID-19	MET-minutes/week during COVID-19	*p*-value	Sedentary hours/day before COVID-19	Sedentary hours/day during COVID-19	*p*-value
BMI category before COVID-19						
Underweight (n = 38)	2997.2 ± 3805.0	2329.3 ± 3465.0	**0.031**	7.5 ± 3.8	8.5 ± 3.9	**0.026**
Normal (n = 131)	3045.8 ± 3581.3	2710.1 ± 3540.1	0.123	6.7 ± 3.5	7.6 ± 3.4	**<0.001**
Overweight (n = 99)	2712.8 ± 3061.1	2494.8 ± 3280.0	0.365	6.1 ± 3.6	7.0 ± 3.9	**<0.001**
Obese (n = 70)	3256 ± 3483.5	2689.8 ± 2926.3	**0.036**	5.6 ± 3.5	5.5 ± 3.8	0.758
Age group						
18–30 (n = 176)	3042.0 ± 3066.4	2499.8 ± 3064.6	**0.007**	7.1 ± 3.5	8.3 ± 3.5	**<0.001**
> 30 (n = 162)	2925.8 ± 3800.1	2709 ± 3592.0	0.175	5.5 ± 3.5	5.7 ± 3.7	0.186

BMI: body mass index; MET: metabolic equivalent.

Paired *t*-test was performed with a significance level of *p* < 0.05.
